# Decrease in Sphingomyelin (d18:1/16:0) in Stem Villi and Phosphatidylcholine (16:0/20:4) in Terminal Villi of Human Term Placentas with Pathohistological Maternal Malperfusion

**DOI:** 10.1371/journal.pone.0142609

**Published:** 2015-11-16

**Authors:** Kaori Yamazaki, Noritaka Masaki, Yukiko Kohmura-Kobayashi, Chizuko Yaguchi, Takahiro Hayasaka, Hiroaki Itoh, Mitsutoshi Setou, Naohiro Kanayama

**Affiliations:** 1 Department of Obstetrics and Gynecology, Hamamatsu University School of Medicine, Hamamatsu, Japan; 2 Department of Cell Biology and Anatomy, Hamamatsu University School of Medicine, Hamamatsu, Japan; 3 Faculty of Health Sciences, Health Innovation & Technology Center, Hokkaido University, Sapporo, Japan; 4 Department of Food and Health Research by NB and LSI, Global Research Center for Food & Medical Innovation, Sapporo, Japan; Montana State University, UNITED STATES

## Abstract

Placental villi play pivotal roles in feto-maternal transportation and phospholipids constitute a major part of the villous membrane. We have been developing and optimizing an imaging system based on a matrix-assisted laser desorption/ionization (MALDI)-based mass spectrometer, which provides clear two-dimensional molecular distribution patterns using highly sensitive mass spectrometry from mixtures of ions generated on tissue surfaces. We recently applied this technology to normal human uncomplicated term placentas and detected the specific distribution of sphingomyelin (SM) (d18:1/16:0) in stem villi and phosphatidylcholine (PC) (16:0/20:4) in terminal villi. In the present study, we applied this technology to nine placentas with maternal or fetal complications, and determined whether a relationship existed between these specific distribution patterns of phospholipid molecules and the six representative pathological findings of placentas, i.e., villitis of unknown etiology (VUE), thrombus, atherosis, chorioamnionitis (CAM), immature terminal villi, and multiple branched terminal villi. In two placentas with the first and second largest total number of positive pathological findings, i.e., five and three positive findings, the specific distribution of SM (d18:1/16:0) in stem villi and PC (16:0/20:4) in terminal villi disappeared. The common pathological findings in these two placentas were atherosis, immature terminal villi, and multiple branched terminal villi, suggesting the possible involvement of the underperfusion of maternal blood into the intervillous space. On the other hand, the number of pathological findings were two or less in the seven other placentas, in which no specific relationships were observed between the differential expression patterns of these two phospholipids in stem and terminal villi and the pathological findings of the placentas; however, the specific distribution pattern of SM (d18:1/16:0) in stem villi disappeared in four placentas, while that of PC (16:0/20:4) in terminal villi was preserved. These results suggested that the absence of the specific distribution of PC (16:0/20:4) in terminal villi, possibly in combination with the absence of SM (d18:1/16:0) in stem villi, was linked to placental morphological changes in response to maternal underperfusion of the placenta.

## Introduction

The placenta is the largest fetal organ and links the mother to the fetus. Placental villi play pivotal roles in the supply of nutrients and oxygen from the mother, thereby enabling proper fetal development and functions [1], and also form a barrier to toxins and infective organisms in order to protect fetal organs [2]. These villi are also the main contributor to the expression of various kinds of bioactive substances that maintain pregnancy, including human chorionic gonadotropin, progesterone, estradiol, estriol, leptin, and resistin [3].

Phospholipids constitute 75% of all lipids and 9% of the dry weight of the human placenta [4], and are mainly composed of phosphatidylcholine (PC), phosphatidylethanolamine, and sphingomyelin (SM) [5]. Phospholipids are the main component of the membranous composition of the villus structure, particularly membrane construction, and this structure is the pivotal location for substance exchange, protection from pathogens, and the secretion of bioactive substances. Phospholipids also play important roles in the placenta under physiological and pathophysiological conditions. A large number of steroid-metabolizing placental enzymes require phospholipids for their activity [6, 7]. Phospholipid complexes were previously reported to function as carriers of amino acids from the maternal to fetal blood [8]. We previously demonstrated that an injection of microvesicles containing phospholipids into pregnant mice caused placental coagulopathy and fetal growth restrictions [9]. Percy et al., extracted lipids from human placentas and reported significant differences in the composition of PC and acylglycerol between placentas from growth-restricted and normally grown newborns [10].

The development of mass spectrometry (MS) technology has revealed numerous patterns of structural modifications to phospholipids after their initial synthesis. Korkes et al., applied time-of-flight (TOF) MS to lipids extracted from human placentas and showed significant changes in the placentas of women with early-onset preeclampsia [11]. However, most of the placenta is composed of heterogeneous placental villi, and an analysis of extracted placental lipids did not provide any information on the physiological and/or pathophysiological significance of structural modifications to or the spatial distribution of phospholipids in the complex placental villous structure.

The recent development of imaging mass spectrometry (IMS) technology has enabled the two-dimensional assessment of structural modifications to phospholipids by coupling mass spectra with simultaneously recorded spatial information in order to obtain the expression patterns of various molecules in the specimens being analyzed [12–16]. In IMS, the coupling of a TOF mass analyzer with either a matrix-assisted laser desorption/ionization (MALDI) or secondary ion mass spectrometry (SIMS) ion source has created a new generation of mass spectrometers suitable for the direct analysis of lipids in mammalian tissues [17, 18]. The introduction of an electrospray ionization source and intermediate-pressure MALDI combined with a linear ion trap imaging system has improved this technology further. MALDI-based IMS has the ability to provide clear two-dimensional molecular identification with highly sensitive MS from a mixture of ions generated on the tissue surface [19]. We applied this technology to an analysis of the gray matter of brains with Alzheimer’s disease [20]. We recently used this technology to examine normal human term placentas and detected the specific distribution of SM (d18:1/16:0) in stem villi and PC (16:0/20:4) in terminal villi [21]. However, the pathophysiological roles of the specific distribution of SM (d18:1/16:0) in stem villi and PC (16:0/20:4) in terminal villi have not yet been elucidated in detail. Phospholipids constitute major parts of the villous membrane. The development of the placental villous tree, from stem villi to the large numbers of terminal villi, is inadequate to maintain appropriate growth and the well-being of the fetus, by exchanging gas and nutrients [22].

Pathological findings based on placental lesions have contributed to a better understanding of how the placenta functions [23, 24]. Placental mal-performance may result in morbidity or even mortality of the mother and/or fetus [23–25]. Roescher et al., performed a systemic review of the literature over the past 18 years and concluded that placental lesions indicated by pathological examinations were one of the main causes of fetal death and also that neonatal complications were associated with placental pathological findings [26]. It is becoming increasingly clear that impaired placental functioning may have major implications for the well-being of neonates [26]. However, the relationship between placental pathological findings and placental lipid profiles, particularly the area-specific villous distribution of phospholipids, currently remains unclear.

Therefore, the first aim of the present study was to determine whether the specific distribution of SM (d18:1/16:0) in stem villi and PC (16:0/20:4) in terminal villi, which we previously reported in uncomplicated term placentas using MALDI-IMS technology [21], was observed in placentas with maternal or fetal complications. The next aim was to clarify if placental pathological findings were associated with the specific villous distribution of these two phospholipids in placentas with maternal or fetal complications.

## Methods

### Approval

The Ethics Committee of the Hamamatsu University School of Medicine approved all procedures (No. 24–265). Written informed consent was obtained after a full explanation of the study.

### Preparation of placental tissue blocks

Placental tissue blocks were obtained immediately after delivery from nine pregnant Japanese women with maternal or fetal complications who delivered during 36–39 weeks of gestation at Hamamatsu University Hospital. Care was taken to minimize blood contamination. Maternal complications were severe gestational hypertension and severe preeclampsia diagnosed according to the American College of Obstetricians and Gynecologists (ACOG) Practice Bulletin [27]. The fetal complication of growth restrictions diagnosed as small for gestational age (SGA) was due to small birth weights of less than the 10th percentile (-1.28 × standard deviation [SD]) according to the means and SDs of the birth weight chart for Japanese newborns by the Japan Pediatric Society [28]. In the pathological examination, four paraffin blocks were systematically obtained from each placenta by systematic random sampling, as previously described [29]. The tissues were placed in 10% formaldehyde (0.1 m sodium cacodylate buffer, pH 7.4), fixed overnight, automatically processed (Tissue-Tek VIP6, Sakura Finetek, Tokyo, Japan), and then embedded manually in paraffin. The tissue-processing protocol consisted of 14 steps (http://www.sakura-americas.com/products/processing/vip-6/). Each block was made vertically from the maternal side to the fetal side and cut into 3-μm-thick sections, followed by hematoxylin and eosin (HE) staining. In the MALDI-IMS analysis, four tissue blocks of approximately one cubic centimeter were obtained from each placenta immediately after birth and the amnion and decidua were bluntly removed, as previously described [21]. These blocks were then snap frozen in liquid nitrogen and stored separately at -80°C in polystyrene tubes until analyzed. The specimens, thus obtained and stored, were stable for an MALDI-IMS analysis, as previously described [21].

### Pathological examination

**Fig 1:**
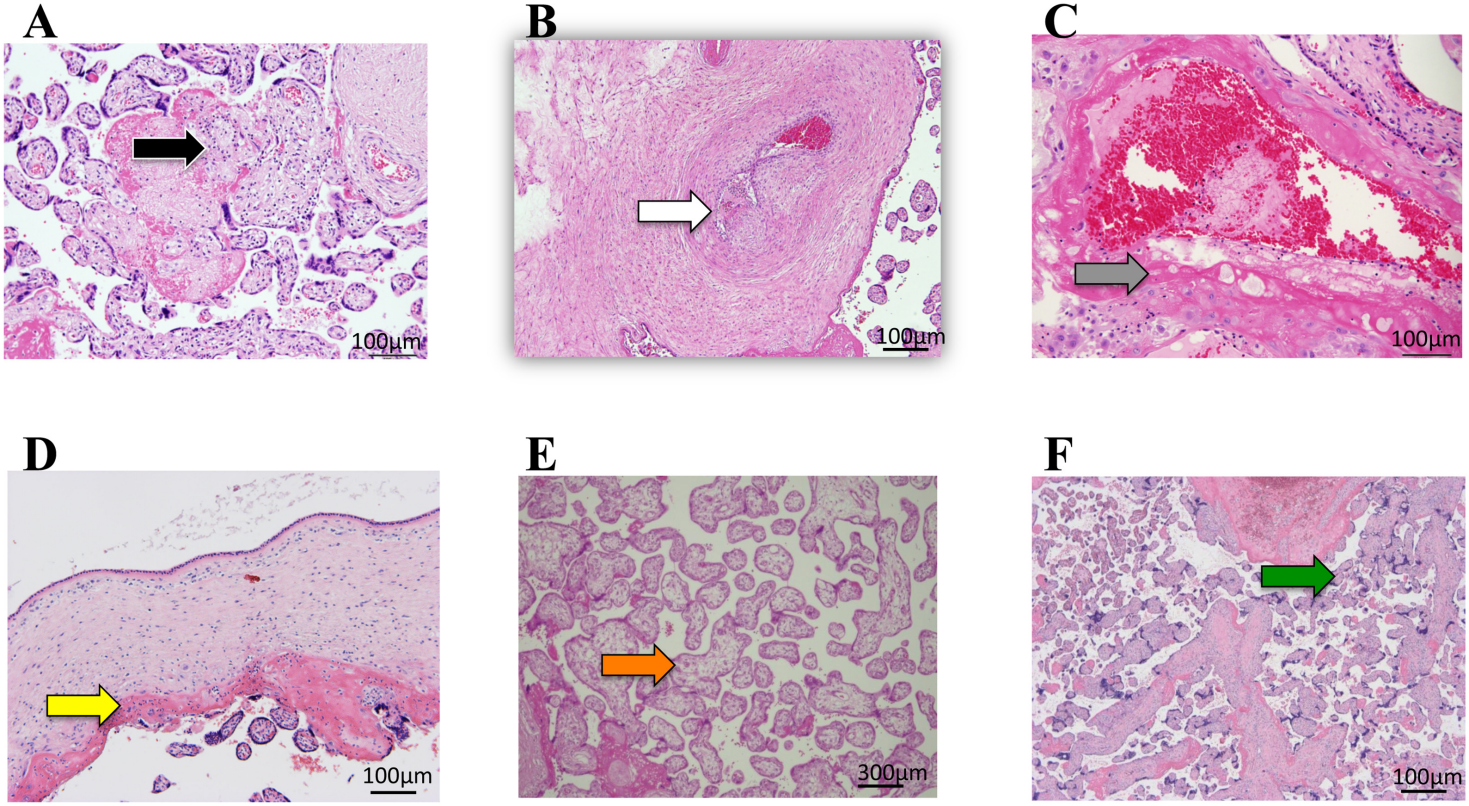
Representative pathological findings by HE staining in placentas with maternal or fetal complications. A; “VUE”; the black arrow indicates lymphohistiocytic inflammation predominantly in the stroma of terminal villi. B; “Thrombus”; the white arrow indicates the endothelial cushion in the walls of the large placental veins. C; “Atherosis”; the gray arrow indicates the replacement of the vessel wall by a fibrin layer. D; CAM; the yellow arrow indicates the infiltration of neutrophils. E; “Immature terminal villi”; the orange arrow indicates increases in the size of distal villi, increases in the numbers of stromal cells, and interstitial fluid uniformly distributed throughout the villous stroma. F; “Multiple branched terminal villi”; the green arrow indicates increases in the numbers of placental villi with the focal formation of tight adherent villous clusters with syncytial knots.

The pathological findings of placentas were classified into the following six categories [25]. “Villitis of unknown etiology (VUE)” is lymphohistiocytic inflammation predominantly localized to the stroma of terminal villi [23–25, 30] (Fig 1A). “Thrombus” was diagnosed as localized, protuberant mural lesions composed of proliferating fibroblasts intermixed with fibrin and erythrocytes in the wall of the large placental vein according to the description of Desa [23–25, 31] (Fig 1B). “Atherosis” is necrotic changes in the vessel wall of decidual spiral arteries, which was diagnosed by the frequent replacement of the vessel wall by a fibrin layer with macrophage infiltration [23–25, 32] (Fig 1C). “Chorioamnionitis (CAM)” was diagnosed by the infiltration of neutrophils into the connective tissues of the chorionic plate and/or the amnion basement membrane in the fetal surface of the placenta, not in the fetal membrane [23–25, 33] (Fig 1D). “Immature terminal villi” was diagnosed as increases in the size of distal villi, increased numbers of stromal cells, and interstitial fluid uniformly distributed throughout the villous stroma [23–25, 34] (Fig 1E). “Multiple branched terminal villi” was diagnosed as increased numbers of placental villi with the focal formation of tight adherent villous clusters [23–25] typically with syncytial knots [35] (Fig 1F). In the present study, each of the six pathological findings was assessed as positive or negative. Each positive pathological finding and the total number of positive pathological findings per placenta were compared with the results of the MALDI-IMS analysis,

### Preparation of samples for the lipid analysis

After being maintained for 30 min at −20°C, a placental tissue block was mounted onto the specimen disc of a cryostat (CM1950; Leica Microsystems, Wetzler, Germany) using optimal cutting temperature (OCT) compound (Sakura Finetek, Torrance, CA). OCT compound was not used to embed the entire tissue because the residual polymer on sections may affect mass spectra. Tissue sections were sliced to a thickness of 8 μm using a cryostat and mounted onto indium tin oxide (ITO)-coated glass slides (Bruker Daltonics, Billerica, MA, USA), as previously described [21]. Three sections were placed on each slide, i.e. a section of a placenta from maternal or fetal complications, a section of a placenta without complications, and a section as an internal control from an identical normal placenta without complications (S1 Fig). Three sections on each slide were analyzed simultaneously. Experiments were conducted repeatedly using the sections obtained from four different blocks per placenta.

### Matrix application

A thin matrix layer was applied to the surface using an airbrush with a 0.2-mm nozzle (Procon Boy FWA Platinum, Mr. Hobby, Tokyo, Japan) [19]. The matrix was sprayed repeatedly for 15 sec at 15-sec intervals (for approximately 15 minutes) until 500 μL of 2,5-dihydroxybenzoic acid (2,5-DHB; Bruker Daltonics) solution (50 mg/mL in 70% methanol/0.1% trifluoroacetic acid) was used. During spraying, the distance between the nozzle and the tissue surface was maintained at 15 cm. Pulsed spraying resulted in the formation of a homogeneous matrix layer on the tissue surface.

### Matrix-assisted laser desorption/ionization imaging mass spectrometry (MALDI-IMS)

**Fig 2:**
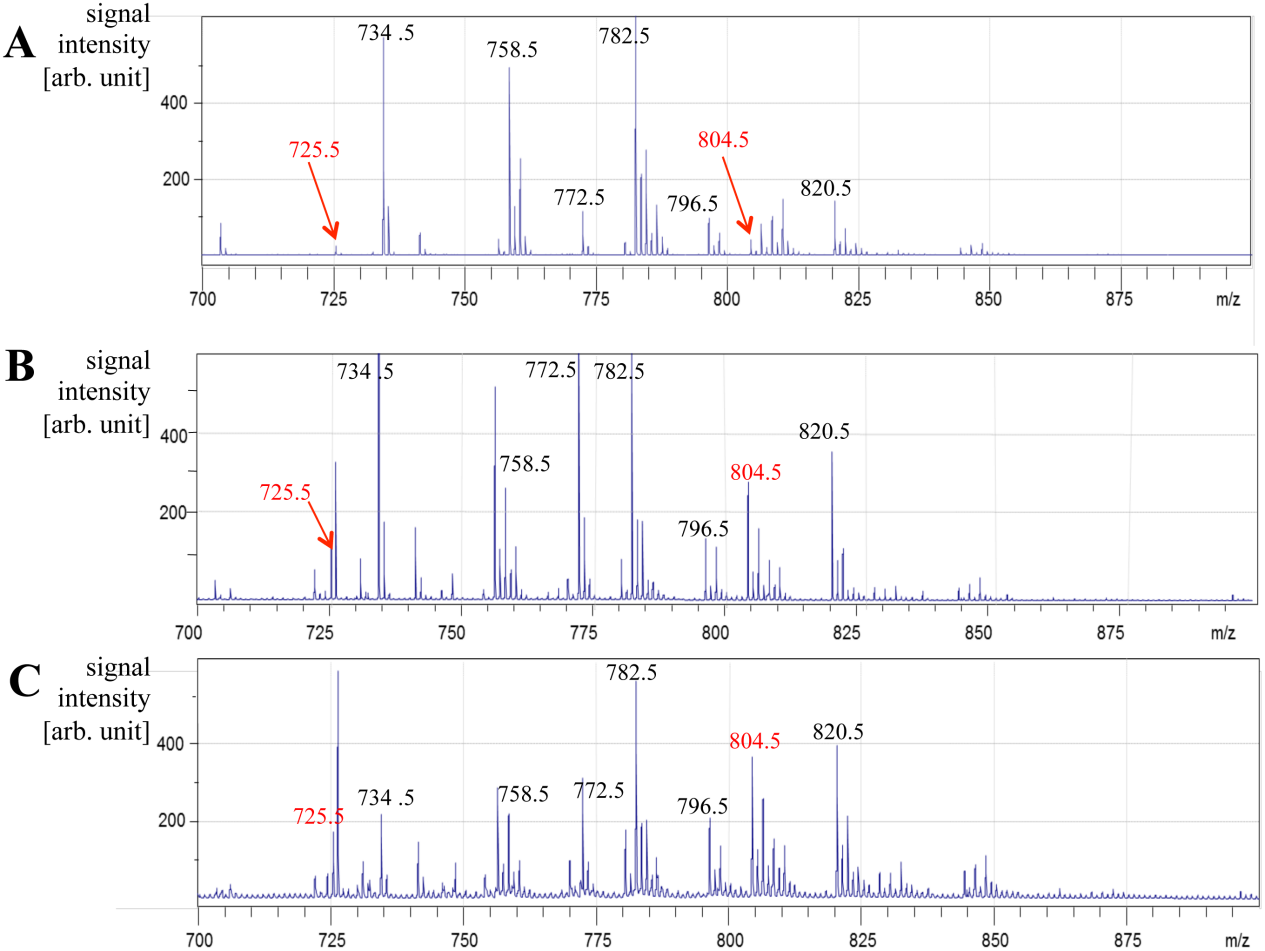
Representative averaged mass spectra from entire sections. Signals collected between *m/z* 700–900 were shown for sample Nos.2 (A), 8 (B), and 9 (C) in Table 3. Peaks corresponding to representative phospholipids were labeled. Imaging results for *m/z* 725.5 and *m/z* 804.5 were shown in Fig 3 and summarized in Table 3. Pathological findings of the placenta were summarized in Table 1.

Immediately after spraying, sections were applied to the conventional desiccator for 3 minutes before the MALDI-IMS analysis. All MALDI-IMS results shown in the present study were acquired using a MALDI-TOF/TOF-type mass spectrometer (ultraflex II; Bruker Daltonics), equipped with a 355 nm Nd:YAG laser [36]. We used a special holder (MTP Slide- Adapter 2; Bruker Daltonics) with concavities to install ITO-coated glass slides in the ionization chamber. All data were acquired in the positive-ion mode using an external calibration method. The external calibration peptides, human angiotensin II ([M + H]^+^, *m/z* 1046.54; Sigma Aldrich, St. Louis, MO) and human bradykinin fragment 1–7 ([M + H]^+^, *m/z* 757.40; Sigma Aldrich), were deposited onto ITO-coated slides. In MALDI-IMS experiments, 200 laser shots per point (a total of 6,550 points) were irradiated using the random walk mode and minimum setting of the Smartbeam Parameter with a repetition rate at 200 Hz and scan pitch of 25 μm. These mass spectra were acquired from each spot with flexControl 3.0 (Bruker Daltonics) (Fig 2). The molecules presented in the present study had been already identified by MS/MS analyses performed on the tissues from normal placentas using the same instrument in our previous study [21]. The human metabolome database (http://www.hmdb.ca/spectra/ms/search) was also referred to for the assignment of other molecules.

### Ion image reconstruction

**Fig 3:**
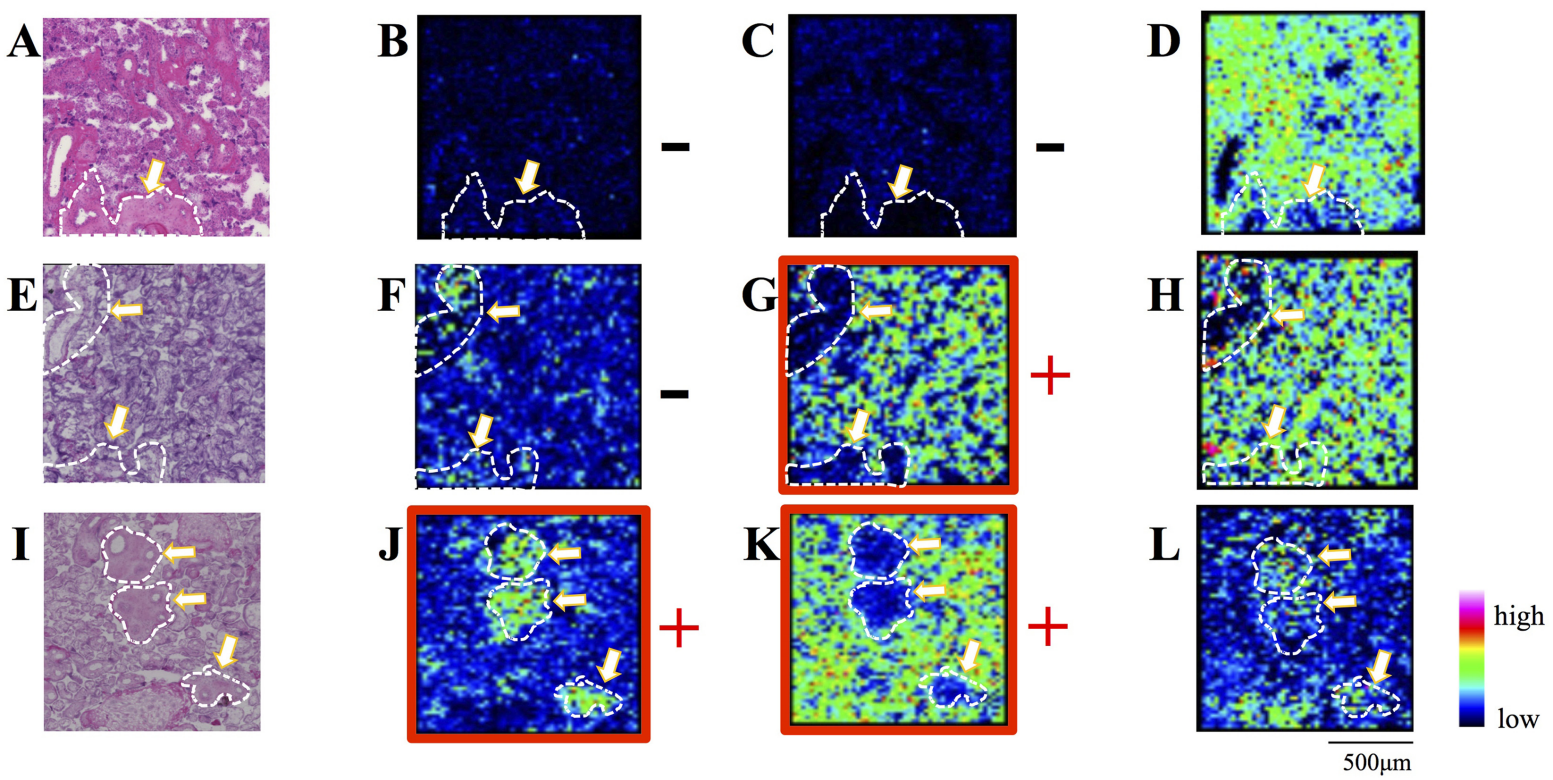
Ion images for *m/z* 725.5 (B, F, J), *m/z* 804.5 (C, G, K), and *m/z* 734.5 (D, H, L). A-D; placenta No. 2. E-H; placenta No.8. I-L; placenta No. 9. The peaks of *m/z* 725.5 corresponding to sphingomyelin (d18:1/16:0) and *m/z* 804.5 corresponding to phosphatidylcholine (16:0/20:4) [21] were visualized. Imaging results for *m/z* 725.5 and *m/z* 804.5 associated with pathological findings were summarized in Tables 1 and 3. An ion image of *m/z* 734.5 (D, H, L) was presented as a positive control independent of pathological findings. A, E, I; HE staining of consecutive sections. Pathological findings of the placenta were summarized in Table 1. The white arrows indicate stem villi. The areas surrounded by white dotted lines correspond to stem villi, in which the specific distribution of *m/z* 725.5 was observed in (J), but not in (B) or (F). The outside areas of white dotted circles are mostly terminal villi, in which the specific distribution of *m/z* 804.5 was observed in (G) and (K), but not in (C). +; Preservation of the specific distribution of *m/z* 734.5 in stem villi (J) or *m/z* 804.5 in terminal villi (G, K). Red squares also indicate the preservation of the specific distribution of *m/z* 734.5 in stem villi (J) or *m/z* 804.5 in terminal villi (G, K). −; Absence of the specific distribution of *m/z* 734.5 in stem villi (B, F) or *m/z* 804.5 in terminal villi (C).

We performed spectrum normalization with the total ion current (TIC) for each mass spectrum (700 < *m/z* < 900)] [37, 38]. TIC normalization is performed automatically with the “Normalize Spectra” function of flexImaging 4.0 software (Bruker Daltonics). The two-dimensional images thus obtained were compared with HE staining of a consecutive section (Fig 3).

### Statistical analysis

We analyzed differences related to pathological changes in the signals of MALDI-IMS data using an analysis of variance (ANOVA) for a single factor as the total number of pathological findings per placenta. The significance of differences between higher and lower total numbers was analyzed using Turkey’s range test. The analytical procedure was as follows; after the export of MALDI-IMS data analyzed in a 7.5 format, the single intensities at each *m*/*z* were extracted and averaged by SIMtools, house made programs in collaboration with Shimadzu (Kyoto, Japan). Statistical analyses were performed for average single intensities with the statics toolbox of MATLAB R2013b (MathWorks, MA, USA). Normalization was not performed for statistical analyses because it could enhance quantitative differences in MALDI-IMS data.

## Results

### Pathological findings of placentas

Table 1 summarized the pathological findings of placentas in a descending order of the total number of positive pathological findings. VUE was observed in placenta No. 1, thrombus in placenta No. 4, atherosis in placenta Nos. 1 and 2, CAM in placenta Nos. 1, 3, and 5, immature terminal villi in placenta Nos.1-3 and 8, and multiple branched terminal villi in placenta Nos. 1, 2, 4–7, and 9.

**Table 1:** Pathological findings in placentas enrolled.

Case	VUE	Thrombus	Atherosis	CAM	Immature Terminal villi	Multiple Branched Terminal villi	Total number of positive pathological findings
1	+	-	+	+	+	+	5
2	-	-	+	-	+	+	3
3	-	-	-	+	+	-	2
4	-	+	-	-	-	+	2
5	-	-	-	+	-	+	2
6	-	-	-	-	-	+	1
7	-	-	-	-	-	+	1
8	-	-	-	-	+	-	1
9	-	-	-	-	-	+	1

VUE; villitis of unknown etiology. CAM; Chorioamnionitis.

Five and three positive pathological findings were observed in placenta Nos. 1 and 2, respectively. Two positive pathological findings were observed in placenta Nos. 3–5. Single pathological findings were observed in placenta Nos. 6–9.

### Backgrounds of placentas analyzed

Table 2 summarized the backgrounds of the placentas analyzed. Gestational ages ranged between 36 and 39 weeks. Two placentas were complicated with maternal severe gestational hypertension. Two placentas were also complicated with maternal severe preeclampsia. The five other placentas were characterized as being small for the gestational age (SGA) as a result of the fetal complication of growth restrictions *in utero*.

**Table 2:** Clinical backgrounds of placentas enrolled in this study.

Case	Age	Gravida Para	BMI (kg/m^2^)	Gestational age	BW (g)	PW (g)	BW/ PW	UmA pH	Maternal complication	Fetal complication
1	43	1G0P	22.2	38w3d	3578	730	5.05	7.347	severe GH	
2	25	3G2P	19.3	39w5d	3688	650	5.50	7.315	severe GH	
3	36	1G1P	26.3	36w2d	2234	470	4.75	7.387	severe PEC	
4	39	1G1P	18.0	36w0d	1372	520	4.60	7.219		SGA(-3.9SD)
5	31	1G1P	22.8	38w1d	2140	420	5.10	7.383		SGA(-2.0SD)
6	26	0G0P	19.5	37w3d	2394	500	4.50	7.327	severe PEC	
7	31	2G1P	20.0	38w0d	2014	360	5.94	7.285		SGA(-2.1SD)
8	43	1G1P	18.9	38w5d	2250	360	3.81	7.295		SGA(-1.5SD)
9	27	0G0P	15.6	37w0d	2254	590	3.82	7.173		SGA(-1.5SD)

BMI; body mass index. BW; birth weight. PW; placental weight. UmA pH; umbilical arterial blood pH, PEC; preeclampsia. GH; gestational hypertension. SGA; small for gestational age.

### Mass Spectrum obtained from placental sections

The time required to complete raster scanning at intervals of 25 μm in 15 mm^2^ was less than three hours. The averaged mass spectrum from raster scanning was shown in the range of *m/z* 700–900, in which many ions representing phospholipids were detected (Fig 2). The two ions *m/z* 725.5 and *m/z* 804.5 were selected and analyzed according to our previous findings on uncomplicated normal term placentas [21].

### Imaging and identification of phospholipids

We visualized two peaks, at *m/z* 725.5 and *m/z* 804.5, because we previously demonstrated that *m/z* 725.5 and *m/z* 804.5 were specifically distributed in the stem and terminal villi of normal uncomplicated term placentas, respectively [21]; however, raster scanning detected a large number of peaks (Fig 2). The *m/z* 725.5 and *m/z* 804.5 peaks were identified as SM (d18:1/16:0) and sodium-ion-adducted PC (16:0/20:4), respectively, by the MS/MS analysis in our previous study [21].

As shown in the HE staining of consecutive sections in Fig 3, stem villi, indicated by white arrows, were surrounded by numerous terminal villi, indicated by yellow arrows, in the analyzed area. The stem villus was identified by the relative size of its area.

Our previous IMS analysis of normal uncomplicated term placentas showed that SM (d18:1/16:0) was distributed in stem villi, but not in terminal villi, and that PC (16:0/20:4) was distributed in terminal villi, but not in stem villi [21]. In the present study, an IMS analysis of term placentas with maternal or fetal complications showed three district patterns according to the distribution of these phospholipids in villi (Table 3). The specific expression patterns of SM (d18:1/16:0) in stem villi and PC (16:0/20:4) in terminal villi were absent in placenta Nos. 1 and 2 (Figs 2A, 3B and 3C) (Table 3) with the two highest total number of positive pathological findings of five and three, respectively (Table 1). The specific expression pattern of SM (d18:1/16:0) was not identified in stem villi, whereas that of PC (16:0/20:4) was preserved in terminal villi in placenta Nos. 3, 6, 7, and 8 (Figs 2B, 3F and 3G) (Table 3) with one or two positive pathological findings (Table 1). In contrast, the specific expression patterns of SM (d18:1/16:0) in stem villi and PC (16:0/20:4) in terminal villi were preserved (Figs 2C, 3J and 3K) (Table 3) in placenta Nos. 4, 5, and 9 with one or two positive pathological findings (Table 1).

**Table 3:** Expression profiles of sphingomyelin (d18:1/16:0) in stem villi and phosphatidylcholine (16:0/20:4) in terminal villi by imaging mass spectrometry.

Case	Elevation in the local expression of sphingomyelin (d18:1/16:0) in stem villi	Elevation in the local expression of phosphatidylcholine (16:0/20:4) in terminal villi
1	-	-
2	-	-
3	-	+
4	+	+
5	+	+
6	-	+
7	-	+
8	-	+
9	+	+

Samples grouped as—showed significant decreases (p<0.05) with an analysis of variance and Tukey’s test from the other group denoted as +.

The loss of MALDI-IMS signals, denoted as ‘-’ in Table 3, compared to preserved cases, denoted as ‘+’, was determined to be significant by an analysis of variance (ANOVA) and Tukey’s test; *p* = 0.02 for SM (d18:0/16:0) and *p* = 0.04 for PC (16:0/20:4). The loss of SM (d18:0/16:0) in stem villi and PC (16:0/20:4) in terminal villi was only observed in placenta Nos.1 and 2, the number of pathological findings of which were above the average in the present study. The signal intensity of *m/z* 772.5, corresponding to PC(32:0)+K, was also significantly decreased in these two samples (*p* = 0.04); however, its expression appeared to be similar in stem and terminal villi (data not shown).

## Discussion

In the human term placenta, terminal villi, particularly syncytiotrophoblasts, are the main compartment for gas and nutrient transport as well as the secretion of placental hormones, whereas stem villi play a pivotal role in supplying fetal blood to numerously branching terminal villi. Our previous IMS analysis of normal uncomplicated term placentas showed that SM (d18:1/16:0) and PC (16:0/20:4) were specifically expressed in stem and terminal villi, respectively, suggesting the possible involvement of these two phospholipids in the morphological and/or functional differentiation of stem and terminal villi as a physiological contribution [21]. However, it currently remains unclear whether this specific distribution of phospholipids in stem and terminal villi is involved in morphological and/or functional abnormalities in placentas with maternal or fetal complications.

In the present study, we determined whether these distinct tissue distribution patterns of SM (d18:1/16:0) in stem villi and PC (16:0/20:4) in terminal villi were associated with morphological abnormalities in placentas with maternal or fetal complications, as indicated by histological examinations. Among the nine placentas examined, the specific distribution of PC (16:0/20:4) in terminal villi disappeared in two placentas, but was preserved in the seven other placentas (Figs 2A, 2B, 2C, 3C, 3G and 3K) (Table 3). These two placentas had the first and second largest number of positive pathological findings of five and three, respectively (Table 1). Three common pathological findings were “atherosis”, “immature terminal villi”, and “multiple branched terminal villi” (Table 1).

The positive finding of “atherosis” coincided with the disappearance of the specific distribution of PC (16:0/20:4) in terminal villi (Tables 1 and 3). “Atherosis” is characterized by the mural hypertrophy of decidual arterioles similar to the changes observed with renal atherosclerosis [23–25] (Fig 1C), and has been reported in some cases of preeclampsia, maternal diabetes, or fetal growth restrictions [24]. Since a previous study on the uterine arcuate artery using Doppler revealed high vascular resistance in placentas with “atherosis” [39], the underperfusion of maternal blood into the intervillous space was suggested in these two placentas. “Multiple branched terminal villi” indicates a higher number of placental villi with the focal formation of tight adherent villous clusters typically concomitant with prominent syncytial knots [23–25, 35] (Fig 1F). Todros et al., performed ultrasound examinations and suggested that “multiple branched terminal villi” may sometimes represent a form of villous adaptation to the underperfusion of maternal blood into the intervillous space by decidual vasculopathy [40]. The combinations of “atherosis” and “multiple branched terminal villi” are frequently, but not always, described in the placental histology of pregnancy-induced hypertension [23–25]. A previous study hypothesized that pregnancy-induced hypertension was causatively linked to spiral arteriopathy in the decidua and some portion of the myometrium induced by insufficient endovascular trophoblast invasion [41]. Therefore, placenta Nos.1 and 2 were complicated with maternal severe gestational hypertension, one of the subcategories of pregnancy-induced hypertension (Table 2). On the other hand, previous studies demonstrated that the decidual vasculopathy of “atherosis” was sometimes causatively associated with “distal villous immaturity” [24, 42]. Taken together, these findings demonstrate that the disappearance of the specific expression of PC (16:0/20:4) in terminal villi in placenta Nos.1 and 2 may occasionally be linked to morphological changes in terminal villi, i.e. multiple branched terminal villi and/or villous immaturity because both of these changes may sometimes be induced in response to the underperfusion of maternal blood into placentas by the decidual vasculopathy of “atherosis”.

Thus, a large number of studies have shown that the pathology of decidual arteries, represented by “atherosis”, is closely connected to the structural as well as functional changes induced in the placental villi by the underperfusion of maternal blood into the intervillous space. [24, 39–41]. The present results of a relationship between disappearances in the specific expression of PC (16:0/20:4) in placental terminal villi and the decidual pathology of “atherosis” also support the concept that decidual vasculopathy is causatively associated with structural changes in placental villi located close to the decidua.

We previously demonstrated that arachidonic acid (AA) contained in PC, PC (16:0/20:4), is characteristic of terminal villi using an MS/MS analysis [21]. AA contained in PC may be the initial substrate in the synthetic cascades of eicosanoids. Lee et al., demonstrated that changes in placental eicosanoid production contribute to impaired vascular remodeling in pregnancy-induced hypertension through insufficient endovascular trophoblast invasion [43]. Insufficient trophoblast invasion was previously hypothesized to be causatively associated with the vasculopathy of decidua and some parts of the myometrium [44], including the pathological lesions of “atherosis” [45]. Nicola et al., reported that prostaglandin E_2_ played pivotal roles in the regulation of trophoblast invasion [46, 47]. Collectively, these findings and the present results suggest that PC (16:0/20:4)-coupled AA is involved in the villus pathological changes associated with the malperfusion of maternal blood into the intervillous space. Therefore, the specific disappearance of the distribution of AA coupling PC (16:0/20:4) in the terminal villi of placentas with “atherosis” and its associated pathological changes in the villous structure may provide an insight into how the malperfusion of maternal blood induces specific changes in the villous structure.

We also noted that the specific distribution of SM (d18:1/16:0) in stem villi also disappeared in these two placentas (Figs 2A and 3B and Table 3). The combination of the disappearance of PC (16:0/20:4) in terminal villi and SM (d18:1/16:0) in stem villi may be linked to the combination of the pathological findings of “atherosis”, “immature terminal villi”, and “multiple branched terminal villi”.

The specific distribution of PC (16:0/20:4) in terminal villi and SM (d18:1/16:0) in stem villi was preserved in three placentas (Nos. 4, 5, and 9) (Figs 2C, 3J and 3K) (Table 3), which was consistent with our previous findings on normal uncomplicated placentas [21]; however, these placentas had one or two positive pathological findings (Table 1), suggesting that the disappearance of the specific distribution patterns of either of the two phospholipids was not inevitable for the development of these pathological findings. On the other hand, the specific expression pattern of SM (d18:1/16:0) in stem villi disappeared in four placentas (Nos. 3, and 6–8), while the specific expression of PC (16:0/20:4) in terminal villi was preserved (Figs 2B, 3F and 3G) (Table 3). These four placentas had one or two positive pathological findings (Table 1). However, there was no specific difference in the pattern of pathological findings between the former (Nos. 4, 5, and 9) and latter placentas (Nos. 3 and 6–8). Thus, we were unable to determine the exact relationship between the specific distribution of PC (16:0/20:4) in terminal villi and SM (d18:1/16:0) in stem villi if the numbers of positive pathological findings were two or less. We presently cannot provide a clear explanation for why three or more positive pathological findings are necessary for the disappearance of the distribution patterns of these specific phospholipids. Therefore, further studies are warranted.

Since anti-phospholipid antibodies have been associated with placental pathology [48], one of our future aims is to investigate the presence of autoimmuno-antibodies specifically against PC (16:0/20:4) or SM (d18:1/16:0) and their possible involvement in the pathology of the placenta.

A limitation of the present study was that we were unable to grant the ubiquitousness of our results in the entire villi of each placenta due to methodological restrictions. However, to the best of our knowledge, a lipid extraction procedure is needed in order to analyze the lipid content of an entire placenta [11], the results of which cannot provide information on the two-dimensional distribution of specific lipids in the complicated structure of villi. Therefore, difficulties are associated with comparing these findings with the histological observations of placentas. The application of MALDI-IMS technology to the investigation of placental villi, even with this slight limitation, may provide an insight into the roles of specific lipids in regulating the structure as well as function of placental villi.

In summary, using MALDI-IMS, we compared the specific distribution of SM (d18:1/16:0) in stem villi and PC (16:0/20:4) in terminal villi with positive pathological findings in human term placentas with maternal or fetal complications. The results obtained suggested that the absence of the specific distribution of PC (16:0/20:4) in terminal villi, possibly in combination with the absence of SM (d18:1/16:0) in stem villi, was linked to the multiple pathological findings in these placentas in association with the malperfusion of maternal blood into the intervillous space.

## Supporting Information

S1 Fig A representative scan image of a sample slide with three sections for an MALDI-IMS analysis as scanned using a flatbed scanner. The left section shows a placenta from maternal or fetal complications. The middle section shows the section of a placenta without complications. The right section is a section as an internal control from an identical normal placenta without complications. Three sections on each slide were analyzed simultaneously.
(PPTX)
