# Supplementary figures and images for: Decrease in Sphingomyelin (d18:1/16:0) in Stem Villi and Phosphatidylcholine (16:0/20:4) in Terminal Villi of Human Term Placentas with Pathohistological Maternal Malperfusion
Source: PLoS One. 2015 Nov 16;10(11):e0142609. doi: 10.1371/journal.pone.0142609 (PMC4646668; doi:10.1371/journal.pone.0142609)

## Slide 1
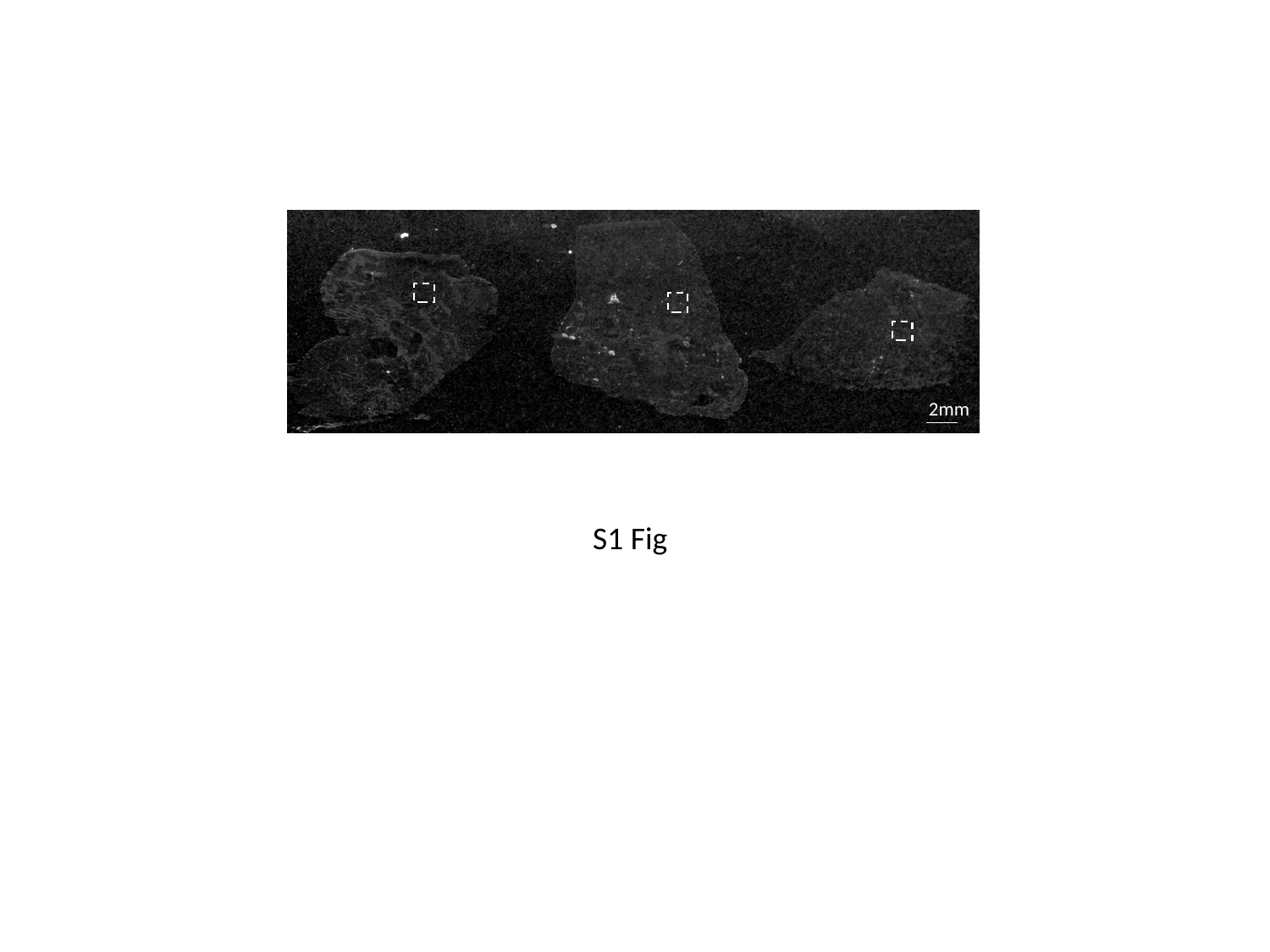

2mm
S1 Fig

Supplement: S1 Fig — The left section shows a placenta from maternal or fetal complications. The middle section shows the section of a placenta without complications. The right section is a section as an internal control from an identical normal placenta without complications. Three sections on each slide were analyzed simultaneously. (PPTX) [file pone.0142609.s001.pptx]
